# Evaluation of HPLC Profile, Antioxidant, Quorum Sensing, Biofilm, Swarming Motility, and Enzyme Inhibition Activities of Conventional and Green Extracts of *Salvia triloba*

**DOI:** 10.1002/fsn3.4580

**Published:** 2024-11-24

**Authors:** Mohammed Mansour Quradha, Alfred Ngenge Tamfu, Mehmet Emin Duru, Selcuk Kucukaydin, Mudassar Iqbal, Abdulkader Moqbel Farhan Qahtan, Rasool Khan, Ozgur Ceylan

**Affiliations:** ^1^ College of Education Seiyun University Seiyun Yemen; ^2^ Pharmacy Department, Medical Sciences Aljanad University for Science and Technology Taiz Yemen; ^3^ Department of Chemical Engineering, School of Chemical Engineering and Mineral Industries University of Ngaoundere Ngaoundere Cameroon; ^4^ Food Quality Control and Analysis Program, Ula Ali Kocman Vocational School Mugla Sitki Kocman University Ula Mugla Turkey; ^5^ Department of Chemistry, Faculty of Science Mugla Sitki Kocman University Menteşe Mugla Turkey; ^6^ Department of Medical Services and Techniques, Koycegiz Vocational School of Health Services Mugla Sıtkı Kocman University Koycegiz Mugla Turkey; ^7^ Department of Agricultural Chemistry and Biochemistry The University of Agriculture Peshawar Peshawar Pakistan; ^8^ Institute of Chemical Sciences University of Peshawar Peshawar Pakistan

**Keywords:** antioxidant, biofilm, conventional extract, enzyme inhibition, green extract, phenolic compounds, quorum sensing, *Salvia triloba*, swarming motility

## Abstract

The current study aims to prepare a green extract using a new method in addition to conventional extraction methods including; methanolic and ultrasonic extraction of *Salvia triloba*, to compare their phenolic composition utilizing high‐performance liquid chromatograph equipped with a diode array detector (HPLC‐DAD), anti‐bacterial, anti‐oxidant, and enzyme inhibition activities. The results of HPLC‐DAD analysis showed that Rosmarinic acid was found the highest amount in the methanolic extract followed by ultrasonic and green extracts as 169.7 ± 0.51, 135.1 ± 0.40, and 28.58 ± 0.46 μg/g respectively. The Trans‐cinnamic acid (4.40 ± 0.09 μg/g) was found exclusively in ultrasonic extract. For bioactivities, the green extract exhibited the highest biofilm inhibition against *Enterococcus faecalis* compared to other extracts, while the methanolic extract outperformed both ultrasonic‐assisted and green extract against *Staphylococcus aureus* and *Escherichia coli* strains at minimum inhibitory concentration. The methanolic and green extract exhibited considerable quorum sensing inhibition against *Chromobacterium violaceum* CV026, while no activity was recorded from ultrasonic‐assisted extract. The methanolic and ultrasonic‐assisted extracts of *S. triloba* recorded moderate butyrylcholinesterase inhibition; each extract demonstrated limited inhibitory effects on the urease enzyme. Similarly, each extract of *S. triloba* demonstrated significant antioxidant activity, with the highest activity exhibited by methanolic extract as β‐carotene‐linoleic acid assay (IC_50_ = 10.29 ± 0.36 μg/mL), DPPH^•^ assay (IC_50_ = 27.77 ± 0.55 μg/mL), ABTS^•+^ assay (IC_50_ = 15.49 ± 0.95 μg/mL), metal chelating assay (IC_50_ = 57.80 ± 0.95 μg/mL), and CUPRAC (assay *A*_0.50_ = 32.54 ± 0.84 μg/mL). Furthermore, the methanolic extract exhibited antioxidant activity better than α‐tocopherol (Standard used). The current study demonstrated the potential of green solvent(s) as eco‐friendly alternative for extractin phenolic compounds from *S. triloba* and evaluated their biological activities for the first time.

## Graphical abstract

The current study demonstrated the ability of the green solvent as a friendly solvent to extract phenolic compounds from *Salvia triloba* and evaluated their biological activities for the first time.

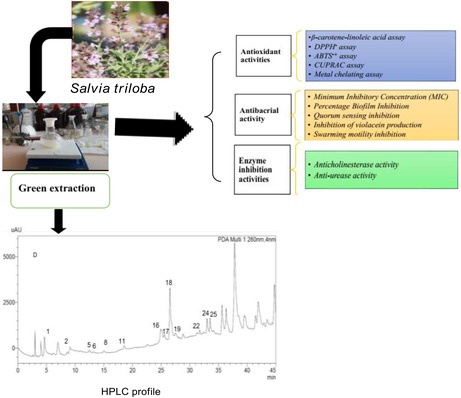

## 1 Introduction

Natural products play a crucial role in drug development due to their structural diversity, and biological activity and as inspiration for synthetic drugs. Their complex and unique chemical structures surpass those synthesized in labs and provide numerous starting points for drug development. Many natural products interact with biological systems, making them potentially medicine; they can also inspire the creation of synthetic drugs with similar properties (Dzobo 2022; Newman and Cragg 2015). Despite advances in synthetic chemistry, natural products continue to provide novel active ingredient and lead structures. Further exploration chemical diversity of the naural world holds promise for the discovery of more effective medicines (Atanasov et al. 2021; Thomford et al. 2018). The first step obtaining bioactive compounds from natural sources for various purposes such as cosmetics, food, and pharmaceuticals is extraction (Calinescu et al. 2023). The bioactive compounds are extracted from natural sources using conventional solvents. However, using conventional solvents to extract bioactive compounds from natural sources has several disadvantage; they can harm the environment through air pollution and toxic waste; health risks include respiratory diseases and cancer. The costs of production and disposal are high; inefficiency leads to the loss of valuable compounds and time‐consuming processes (Dai, Witkamp, et al. 2013; Chemat et al. 2019; Paiva et al. 2014). Researchers are seeking for alternative methods to address these problems. Green solvents are derived from renewable sources, reducing the need for fossil fuels. They are less toxic and flammable than traditional solvents, creating a safer workplace. Green solvents offer better selectivity to extract specific compounds from natural sources for improved products (Marinaccio et al. 2024; Clarke et al. 2018; Peng et al. 2015; Shishov et al. 2017; Wei et al. 2015). *Salvia triloba* is medicinal plant, traditionally known as Greek sage; traditionally used to cure headaches and improve memory (Abdelhalim et al. 2014) and used as antipyretic, diuretic, and antiseptic and for wound healing (El‐Sayed et al. 2001); it is also reported for antibacterial and antioxidant (Paula et al. 2007; Yıldırım and Mavi 2000), antiproliferative (Abu‐dahab et al. 2015), antiinflammatory, and ulcerogenic (El‐Sayed et al. 2006) properties. Many pathogenic bacteria are able to sense changes in their environment and tend to change their behavior to adapt and overcome adversities such as presence of antibiotics, drought, starvation, and host the immune system through various means and using virulence factors (Benaissa et al. 2024; Lazar et al. 2021). The most difficult situation concerns the ability of bacteria to move from free floating planktonic colonies to adherent enveloped colonies called biofilms using various motilities like swarming, swimming, and twitching with the transition from motility‐to‐biofilm transition promoting biofilm formation (Doğaç et al. 2024; Guttenplan and Kearns 2013). Biofilms consist of a dense, protective, and impermeable membrane composed largely of self‐made extracellular polymeric substances produced through cell‐to‐cell communication networks mediated by quorum sensing (QS) and serves as an adaptive strategy (Tamfu et al. 2022). Biofilm infections are challenging to treat since they are not exposed to antibiotics, stress factors, and host defense system. Biofilms, quorum‐sensing, swarming, and swimming motilities are among the virulence factors that lead to the severity of infections and the development of resistance to antibiotics. Inhibiting them can be a good strategy to overcome various infections (Paluch et al. 2020; Tamfu et al. 2023). There is an increasing need to the discover new antimicrobial (AM) materials and substances, particularly those derived from natural sources such as plants that can inhibit QS, biofilm formation, and bacterial motility, among other virulence factors. (Bhattacharya et al. 2023; Ikome et al. 2023; Shariati et al. 2024). Most biochemical processes in living systems involve enzymes, and an imbalance in enzyme activity can lead to pathological conditions such as Alzheimer's disease and hyperpigmentation (Geronikaki 2020; Munvera et al. 2023). Ailments resulting from over‐expression of enzymes can be corrected using enzyme inhibitors. Alzheimer's diseases affects approximately 25 million people worldwide and is a severe neurodegenerative disease in multiple brain regions that lead to acetylcholine deficiency, memory impairment, and progressive brain degradation affecting the patient's daily activities (Tamfu et al. 2021). The enzyme urease plays an important role in the rapid hydrolysis of urea to ammonia and is vital for the sustained colonization of *Helicobacter pylori*. This bacterium is the causative agent of gastrointestinal diseases, including gastritis, duodenal ulcers, peptic ulcers, and stomach cancer (Mahernia et al. 2015). Urease and cholinesterase inhibitors are important in treating ulcers and symptoms of demntia. The contribution of oxidative stress to the above and many others diseases is remarkable. Oxidative stress occurs when reactive oxygen species (ROS), reactive nitrogen species (RNS), as well as various other free radical species accumulate cells, causing a number of diseases including ulceration, diabetes, and Alzheimer's disease (AD) (García‐Sánchez, Miranda‐Díaz, and Cardona‐Muñoz 2020; Reddy 2023). This study is the first time to evaluate and compare the phenolic profiles and potency of conventional extract and green extract of *S. triloba* using HPLC profiling and bioassays. The AM properties of the extracts were evaluated, along with their impact on inhibition of biofilm formation, violacein production, and swarming motility in pathogenic bacteria. The extracts were examined for their ability to inhibit cholinesterase and urease as well as their antioxidant activity.

## 2 Materials and Methods

### 2.1 Collection of Plant

The plant was collected according to the protocol described by Ernst (1995). Aerial parts of *S. triloba* were randomly collected from Akyaka, Ula‐Muğla in Turkey. The herbarium specimen is stored in the Natural Products Laboratory at Muğla Sıtkı Koçman University, Faculty of Science, under the code MUST1024. The collected sample was dried in the shade, ground, and then used for extraction.

### 2.2 Extraction Procedures

#### 2.2.1 Methanolic Extraction

Ten grams of aerial parts of *S. triloba* were macerated in 300 mL of methanol (MeOH) for 72 h at room temperature. The extract was filtered through 0.45‐μm cellulose filter paper, and the rotary evaporator (Heidolph, Hei‐Vap Value G1, Germany) under reduced pressure was utilized for removing methanol. The dry extract obtained was a brownish‐green gummy material with a characteristic camphoraceous odor.

#### 2.2.2 Ultrasonic‐Assisted Extraction

An ultrasonic cleaner (Model Lab. Ult.4030), equipped with the Q600 a processor unit (500 W, 40 kHz), was used for the extraction process. Ten grams of the aerial parts of *S. triloba* were placed in a 500 mL conical beaker, and 300 mL of methanol was added. The beaker was then sealed, and extraction was carried out for 25 min. After extraction, the solution was filtered through 0.45 μm cellulose filter paper, and the resulting extract was concentrated under reduced pressure using a rotary evaporator.

#### 2.2.3 Green Extraction

##### 2.2.3.1 Preparation of Natural Deep Eutectic Solvents (NADES)

The green solvent was prepared using a natural deep eutectic solvent (NADES) consisting of citric acid and glucose, formulated according to previously established protocols (Dai, van Spronsen, et al. 2013; Kumar, Parikh, and Pravakar 2016). Briefly, citric acid and glucose were combined in a molar ratio of 1:2 and placed in a sealed 250 mL Erlenmeyer flask. The mixture was then heated to 80°C under constant stirring until a homogeneous liquid was formed.

##### 2.2.3.2 Crude Extraction Using the Green Solvent

Green extraction was performed by adding the green solvent to 3 g of plant material in a sealed conical flask, followed by adding 20 mL of distilled water to reduce the viscosity. The mixture was heated to 40°C and stirred for 10 h. The sample was then transferred to a 50 mL Falcon tube and centrifuged at 4000 rpm for 20 min. The residues were discarded, and the supernatant was filtered through a 0.45 μm cellulose paper. After filtration, a portion of the solvent was evaporated using a rotary evaporator, while the remaining aqueous part was freeze‐dried and stored at +4°C for further analysis.

### 2.3 Determination of Phenolic Composition

A Shimadzu 20 AT series high‐performance liquid chromatograph equipped with a diode array detector (HPLC‐DAD) (Shimadzu Corporation, Kyoto, Japan) was utilized to analyze the phenolic compounds. The extracts were dissolved in a mixture of water and methanol (80:20) and subsequently filtered through a 0.20 μm disposable LC filter disk to remove suspended particles before being transferred to an Intersil ODS‐3 reverse phase C18 column for separation and detection (Beddiar et al. 2021). The solvent flow rate was set to 1.0 mL/min, and the sample injection volume was 20 μL. The mobile phase comprised 0.5% acetic acid in water as mobile phase A and 0.5% acetic acid in methanol as mobile phase B. The elution gradient was programmed as follows: 0%–10% B (0–0.01 min); 10%–20% B (0.01–5 min); 20%–30% B (5–15 min); 30%–50% B (15–25 min); 50%–65% B (25–30 min); 65%–75% B (30–40 min); 75%–90% B (40–50 min); and 90%–10% B (50–55 min). Detection occurred at a wavelength of 280 nm. The phenolic compounds were identified by comparing their UV data and retention times with those of commercial standards. Quantification was performed using a calibration curve constructed from known concentrations of standard compounds. A total of 26 standard phenolic compounds were analyzed, including gallic acid, protocatechuic acid, chlorogenic acid, *p*‐hydroxy benzoic acid, caffeic acid, 3‐hydroxy benzoic acid, syringic acid, *p*‐coumaric acid, ferulic acid, ellagic acid, rosmarinic acid, trans‐cinnamic acid, catechin, pyrocatechol, 6,7‐dihydroxy coumarin, vanillin, taxifolin, coumarin, rutin, myricetin, quercetin, luteolin, hesperetin, kaempferol, apigenin, and chrysin. The results are expressed in μg/g of dry weight.

### 2.4 Biological Activities

#### 2.4.1 AM Activity

The microorganisms used in the current study are *Staphylococcus aureus* ATCC 25923, *Enterococcus faecalis* ATCC 29212, *Escherichia coli* ATCC 25922, *Candida albicans* ATCC 10239, *Chromobacterium violaceum* CV12472, *Chromobacterium violaceum* CV026, and *Pseudomonas aeruginosa* PA01.

##### 2.4.1.1 Determination of Minimum Inhibitory Concentrations

Minimum inhibitory concentrations (MICs) were determined using the microtite broth dilution method as outlined by the Clinical Laboratory Standards Institute (2006). The MIC was defined as the lowest concentration of the extract that resulted in no visible bacterial growth. Mueller‐Hinton broth served as the test medium, with a bacterial density of 5 × 10^5^ colony‐forming units (CFU)/mL. A volume of 100 μL of cell suspension was added to the 96‐well microtitre plates containing the extracts at various final concentrations (1, 0.5, 0.25, 0.125, 0.0625, and 0.0312 mg/mL). The inoculated plates were incubated at 37°C for 24 h before evaluation.

##### 2.4.1.2 Effect of Extract on Bacterial Biofilm Formation

The impact of *S. triloba* extract(s) at concentrations of 1, 1/2, 1/4, and 1/8 MIC on biofilm formation of test microorganisms was evaluated using a microplate biofilm assay (Merritt et al. 2005). Briefly, 1% of overnight cultures of the isolates were introduced into 200 μL of fresh tryptose‐soy broth (TSB) supplemented with 0.25% glucose and incubated for 48 h at 37°C, both in the presence and absence of the extract(s), without agitation. The control wells contained only TSB+ cells. After incubation, the wells were rinsed with water to eliminate planktonic bacteria. The remaining adherent bacteria were then stained with a 0.1% crystal violet solution for 10 min at room temperature. The wells were washed again to remove excess crystal violet. Subsequently, 200 μL of a 33% glacial acetic acid solution in ethanol was added to each well. After shaking and pipetting, 125 μL of the solution from each well was transferred to a sterile tube, and the volume was adjusted to 1 mL with distilled water. Finally, the optical density (OD) of each well was measured at 550 nm using a Thermo Scientific Multiskan FC (Vantaa, Finland). The percentage inhibition of the tested extracts was calculated using the following formula:

$$\text{Biofilm inhibition}\;\left(\%\right)=\frac{\mathrm{OD}550_{\text{Control}}-\mathrm{OD}550_{\text{Sample}}}{\mathrm{OD}550_{\text{Control}}}\times 100$$

##### 2.4.1.3 Bioassay for Quorum‐Sensing Inhibition (QSI) Activity Using *C. violacium* CV026

The QS inhibition of each *S. triloba* extract was evaluated using a modified version of the method described previously (Koh and Tham 2011), with tiny modifications. A warm mixture of 5 mL of soft top agar (consisting of 1.3 g agar, 2.0 g tryptone, 1 g sodium chloride, and 200 mL deionized water) was inoculated with 100 μL of an overnight culture of *C. violaceum* CV026. To this, 20 μL of a 100 μg/mL solution of C_6_HSL was added as an exogenous AHL source. The mixture was gently mixed and immediately poured over a solidified LBA plate to create an coating. After overlay hardened well with a diameter of 5 mm were formed on each plate. Each well was then filled with 50 μL of filter‐sterilized extracts concentrations below the MIC. The presence of a white or cream‐colored halo surrounding the well against the purple lawn of activated *C. violaceum* CV026 suggested quorum sensing inhibition (QSI), while a clear halo indicated AM activity. The limit of detection for activity was determined by serial dilutions of the extracts (1:1 to 1:8) using LB broth as the diluent, with endpoints defined as the lowest dilution that produced appreciable inhibition of violacein synthesis. Each experiment was performed in triplicate, and the test plates were incubated at 30°C for 3 days, after which the diameters of the QSI zones were measured.

##### 2.4.1.4 Violacein Inhibition Assay Using *C. violacium* CV12472

Extract(s) of the *S. triloba* were subjected to qualitative analysis to find their QSI potentials against *C. violaceum* ATCC 12472 (Tamfu et al. 2020). An overnight culture (10 μL) of *C. violaceum* (10 μL), adjusted to OD 0.4 at 600 nm, was added into sterile microtiter plates containing 200 μL of LB broth and incubated in the presence and absence of sub‐MICs of the extract. The LB broth with *C. violaceum* ATCC 12472 was used as a positive control. These plates were incubated at 30°C for 24 h and observed for the reduction in violacein pigment production. Absorbance measurements were performed at 585 nm and the percentage of *violacein* inhibition was calculated using the following formula:

$$\begin{array}{cc} \text{Violacein inhibition}\;\left(\%\right)= & \;\left(\mathrm{OD}585_{\text{control}}-\mathrm{OD}585_{\text{sample}}\right)/\left(\mathrm{OD}585_{\text{control}}\right)\\ & \;\times 100 \end{array}$$

##### 2.4.1.5 Swarming Motility Inhibition on *P. aeruginosa* PA01

The warming motility inhibition assay of *S. triloba* extracts was evaluated as described previously (Packiavathy et al. 2012). Briefly, overnight cultures of the *P. aeruginosa* PA01 strain were inoculated in the center of swarming plates containing 1% peptone, 0.5% NaCl, 0.5% agar, and 0.5% filter‐sterilized d‐glucose, as well as various extract concentrations (50, 75, and 100 μg/mL). A plate without any extract served as a control. The plates were incubated upright at an appropriate temperature for 18 h. Swarming migration was monitored by observing the movement of the bacterial cell swarm fronts.

#### 2.4.2 Anticholinesterase Activity

The inhibitory activities of acetylcholinesterase (AChE) and butyrylcholinesterase (BChE) by *S. triloba* extract(s) were determined using a spectrophotometer following the protocol described by Ellman, with slight modifications (Öztürk et al. 2014; Stefanucci et al. 2020). Galantamine was used as a reference compound. The IC_50_ values were calculated using a program derived from the anticholinesterase graph. Percentages of inhibitory activity (% inhibition) were developed relative to sample concentrations (μg/mL).

#### 2.4.3 Anti‐Urease Activity

The abilty of *S. triloba* extracts to inhibit urease enzyme was evaluated by measuring ammonia production using the indophenol method (Weatherburn 1967) using with a microplate reader. Briefly, 25 μL of urease solution (derived from jack beans), 50 μL of urea (100 mM), and 100 mM sodium phosphate buffer (pH 8.2) were combined and incubated at 30°C for 15 min after adding 10 μL of the sample extract. Then, 70 μL of alkaline reagent and 45 μL of phenol reagent were added to each well. After an incubation period of 50 min, the absorbance was measured at 630 nm using a microplate reader. Thiourea served as a reference compound, and the results are expressed as 50% inhibitory concentration (IC_50_).

#### 2.4.4 Antioxidant Activities

The antioxidant activities of *S. triloba* (extracts) were measured using various methods including (1) β‐carotene‐linoleic acid assay, (2) DPPH assay, (3) ABTS assay, (4) cupric reducing antioxidant capacity (CUPRAC) assay, and (5) Metal chelating assay. Inhibition of lipid peroxidation activity was performed using the β‐carotene‐linoleic acid test system according to the standards protocol developed by Marco (1968), with minor modifications (Tel‐Çayan and Duru 2019). The DPPH assay was performed by using a spectrophotometer according to typical methods documented previously (Blois 1958; Çayan et al. 2014). The ABTS^+^ assay was performed as previously described by Marco (1968), with slight modification (Tel et al. 2012). The CUPRAC was evaluated according to the published method (Apak et al. 2004). α‐Tocopherol and butylated hydroxyanisole (BHA) were used as antioxidant standards to compare the β‐carotene‐linoleic acid, DPPH, ABTS^+^, and CUPRAC assays. The metal chelating assay of extracts for Fe^+2^ was carried out using a spectrophotometer according to the method described previous (Decker and Welch 1990). Ethylenediaminetetraacetic acid (EDTA) was used as a standard. The results of an antioxident assays were reported as 50% inhibitory concentration (IC_50_).

### 2.5 Statistical Analysis

All activities in the current study were performed in triplicate, and results are presented as the mean ± standard error of the mean. Student's *t*‐test was used to evaluate significant differences between means, with *p*‐values less than 0.05 considered significant. Additionally, all methods presented in this manuscript are confirmed to be in accordance with relevant guidelines and regulations and are appropriately cited as needed.

## 3 Results

Three types of extracts were prepared, the conventional extract was prepared by using methanol and ultrasonic extracts. While green extract was prepared using the new methods; all extracts were subjected to examination and comparison of their HPLC phenolic profile as well as their potential biological activities.

### 3.1 Phenolic Compositions

**FIGURE 1:**
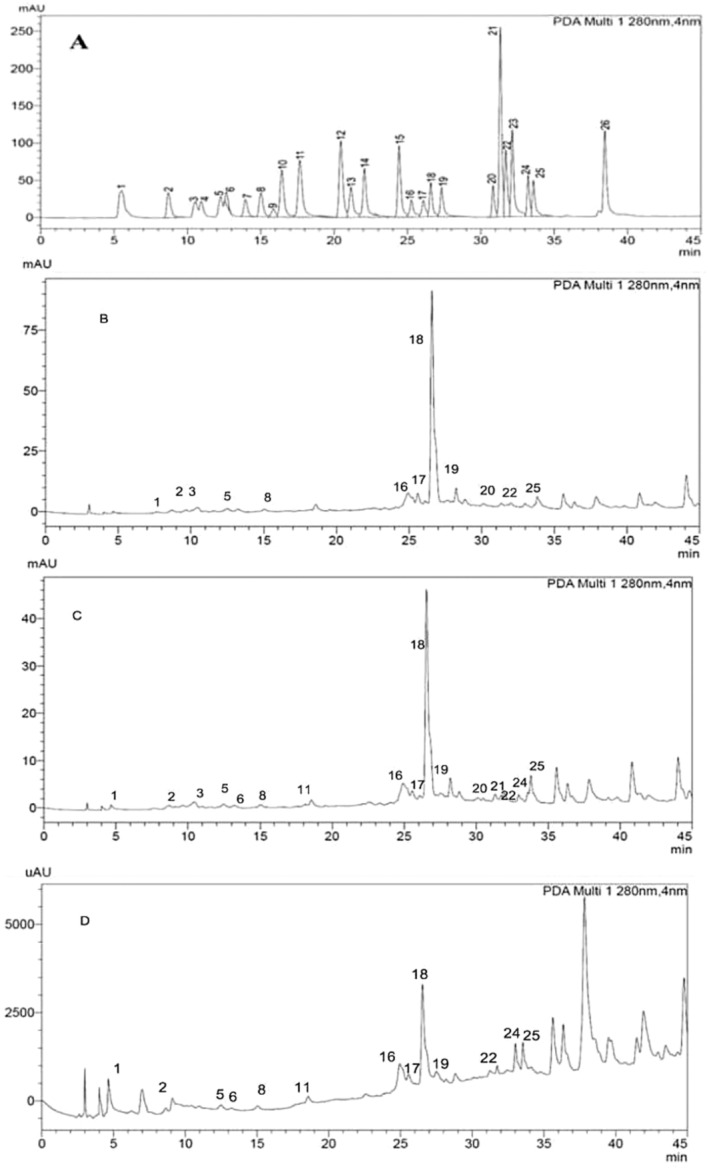
HPLC chromatograms of phenolic compounds, (A) standard phenolics, (B) methanolic extract, (C) ultrasonic extract and (D) green extract.

**FIGURE 2:**
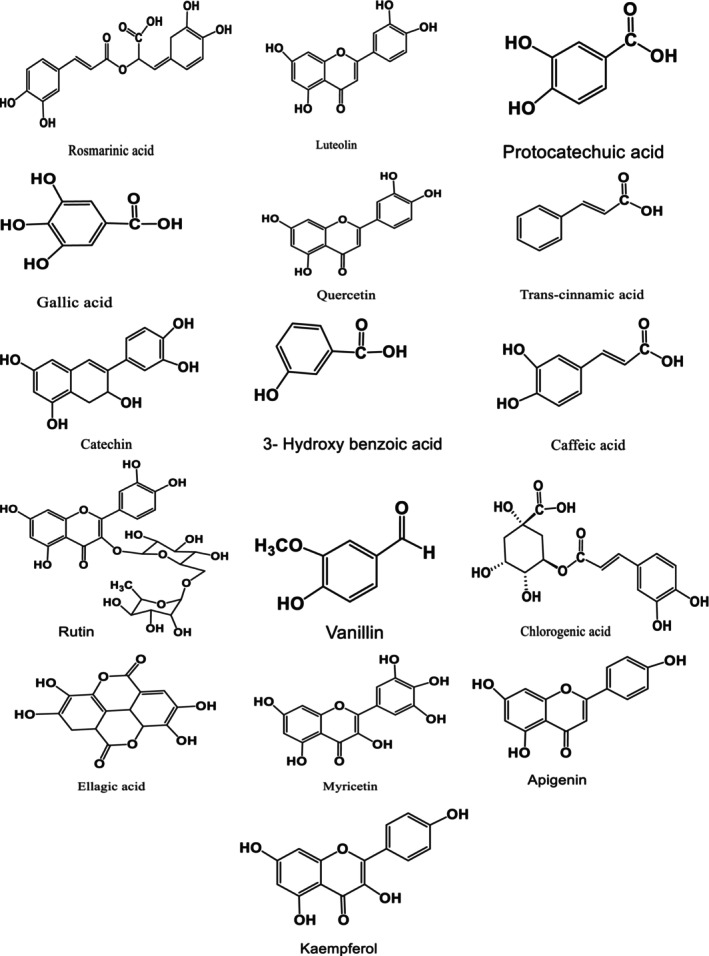
Structures of compounds identified in methanol ultrasonic and green extracts.

The existences of the phenolic composition in green extract and conventional extract represented by (methanol and ultrasonic extracts) of *S. triloba* were detected and measured by HPLC‐DAD. Twenty‐six reference phenolic compounds were used as standards. The results are described in Table 1 and show that the two phenolic compounds comprising the Rosmarinic acid and Rutin were found in high amounts in the methanolic extract quantified as 169.7 ± 0.51 and 21.36 ± 0.31 μg/g respectively, while in ultrasonic extracts as 135.1 ± 0.40 and 21.17 ± 0.27 μg/g respectively, and in green extract, the same phenolic compounds were detected as 28.58 ± 0.46 and 9.35 ± 0.17 μg/g, respectively. The phenolic compounds including gallic acid, protocatechuic acid, chlorogenic acid, caffeic acid, rutin, ellagic acid, rosmarinic acid, myricetin, and apigenin were found in different levels in each tested extract; while catechin and quercetin were also found in methanol extract and ultrasonic extracts. The phenolic compounds including *p*‐hydroxy benzoic acid, vanillin, and kaempferol were found in ultrasonic and green extracts. The trans‐cinnamic acid was found exclusively in ultrasonic extract quantitated as 4.40 ± 0.09 μg/g. The remaining compounds including pyrocatechol, 6,7‐dihydroxy coumarin, 3‐hydroxy benzoic acid, syringic acid, *p*‐coumaric acid, taxifolin, ferulic acid, coumarin, and chrysin were not identified in any of the extract. The HPLC‐DAD chromatograms of green, ultrasonic, and ethanolic extracts of *S. triloba* are presented in Figures 1 and 2.

**TABLE 1:** Phenolic composition of the extracts of *S. triloba* by HPLC‐DAD (μg/g)^a^.

No	Phenolic compounds	RT (min)	Methanol extract	Ultrasonic extract	Citric acid:glycose
1	Gallic acid	5.70	4.58 ± 0.16	3.78 ± 0.11	6.25 ± 0.33
2	Protocatechuic acid	8.75	5.63 ± 0.21	3.55 ± 0.24	3.54 ± 0.18
3	Catechin	10.18	7.54 ± 0.18	7.80 ± 0.30	—
4	Pyrocatechol	11.04	—	—	—
5	Chlorogenic acid	12.35	3.38 ± 0.20	3.20 ± 0.15	2.55 ± 0.14
6	*p*‐Hydroxy benzoic acid	12.77	—	3.40 ± 0.12	2.73 ± 0.20
7	6,7‐Dihydroxy coumarin	14.10	—	—	—
8	Caffeic acid	15.09	4.50 ± 0.11	4.63 ± 0.14	3.65 ± 0.16
9	3‐Hydroxy benzoic acid	15.98	—	—	—
10	Syringic acid	16.56	—	—	—
11	Vanillin	17.78	—	5.73 ± 0.16	4.40 ± 0.25
12	*p*‐Coumaric acid	20.56	—	—	—
13	Taxifolin	21.26	—	—	—
14	Ferulic acid	22.14	—	—	—
15	Coumarin	24.49	—	—	—
16	Rutin	25.30	21.36 ± 0.31	21.17 ± 0.27	9.35 ± 0.17
17	Ellagic acid	26.11	9.34 ± 0.24	7.23 ± 0.15	3.88 ± 0.34
18	Rosmarinic acid	26.77	169.7 ± 0.51	135.1 ± 0.40	28.58 ± 0.46
19	Myricetin	27.35	6.21 ± 0.18	5.81 ± 0.11	5.22 ± 0.32
20	Quercetin	30.83	3.15 ± 0.12	3.49 ± 0.16	—
21	*Trans*‐cinnamic acid	31.33	—	4.40 ± 0.09	—
22	Luteolin	31.70	4.24 ± 0.13	4.82 ± 0.19	4.21 ± 0.10
23	Hesperetin	32.14	—	—	—
24	Kaempferol	33.21	—	3.55 ± 0.20	6.79 ± 0.28
25	Apigenin	33.77	5.21 ± 0.10	9.50 ± 0.25	7.33 ± 0.16
26	Chrysin	38.40	—	—	—

*Note:* —, not detected.

^a^ Values expressed are means ± SEM of three parallel measurements (*p* < 0.05).

#### 3.1.1 Minimum Inhibitory Concentration

**FIGURE 3:**
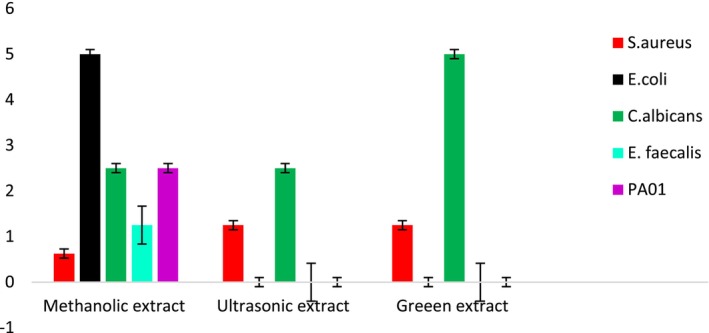
Antimicrobial activity (MIC values in mg/mL) of methanol, ultrasonic and green extracts of *Salvia triloba*.

The current investigation estimated the MIC of methanolic, ultrasonic, and green extracts of *S. triloba* against several bacterial strains, including; *S. aureus*, *E. coli*, *C. albicans*, *E. faecalis*, and *PA01*. The results are summarized in Table 2 and illustrated in Figure 3. The methanolic extract showed considerable AM activity with an MIC of 0.625 mg/mL against *S. aureus*, surpassing the ultrasonic and green extracts, which had MIC values of 1.25 mg/mL. Furthermore, the methanolic extract showed good activity against *E. faecalis*, *C. albicans*, and *PA01*, with MIC values of 1.25, 2.5, and 2.5 mg/mL, respectively. However, it exhibited limited effectiveness against *E. coli*, with an MIC of 5 mg/mL. In contrast, the ultrasonic and green extracts exhibited moderate and weak activity against *C. albicans*, with MIC values of 2.5 and 5 mg/mL, respectively, and no inhibitory effect was observed against *E. coli*, *E. faecalis*, or *PA01*.

**TABLE 2:** Antimicrobial activity of methanol, ultrasonic‐assisted and green extracts of *S. triloba*.

Microorganisms	Methanol extract	Ultrasonic extract	Green extract
*S. aureus*	0.625	1.25	1.25
*E. coli*	5	> 5	> 5
*C. albicans*	2.5	2.5	5
*E. faecalis*	1.25	> 5	> 5
*PA01*	2.5	> 5	> 5

#### 3.1.2 Percentage Biofilm Inhibition

The biofilm inhibition of each extract against selected pathogens was evaluated at both MIC and sub‐MIC concentrations, as shown in Table 3. The results showed that the methanolic extract of *S. triloba* was more effective than the ultrasonic‐assisted and green extracts in inhibiting biofilm formation on *S. aureus* and *E. coli*. In particular, the methanolic extract achieved an inhibition of 49.97 ± 0.85% and 26.7 ± 0.25% against *S. aureus* at MIC and MIC/2 concentrations, respectively, and 42.38 ± 0.82% and 25.12 ± 0.38% against *E. coli* at the same concentrations. Conversely, the green extract demonstrated superior biofilm inhibition compared to the methanolic and ultrasonic extracts on *E. faecalis*, with recorded inhibition rates of 45.31 ± 1.35%, 21.23 ± 0.74%, and 6.1 ± 0.2% at MIC, MIC/2, and MIC/4 concentrations, respectively. In contrast, the ultrasonic‐assisted extract showed better activity against *P. aeruginosa PA01* at MIC, MIC/2, and MIC/4 concentrations, with percentage inhibitions of 31.4 ± 0.47%, 15.3 ± 0.25%, and 4.1 ± 0.02%, respectively.

**TABLE 3:** Anti‐biofilm inhibition of methanol, ultrasonic and green extracts of *S. triloba*.

Microorganisms	MIC (mg/mL)	Methanol extract	Ultrasonic extract	Green extract
*S. aureus*	MIC	49.97 ± 0.85	24.41 ± 0.67	11.55 ± 0.31
	MIC/2	26.7 ± 0.25	9.97 ± 0.08	—
	MIC/4	13.16 ± 0.22	—	—
	MIC/8	—	—	—
*E. coli*	MIC	42.38 ± 0.82	—	—
	MIC/2	25.12 ± 0.38	—	—
	MIC/4	—	—	—
	MIC/8	—	—	—
*C. albicans*	MIC	10.93 ± 0.09	22.84 ± 0.65	—
	MIC/2	—	—	—
	MIC/4	—	—	—
	MIC/8	—	—	—
*E. faecalis*	MIC	30.87 ± 0.71	37.5 ± 0.45	45.31 ± 1.35
	MIC/2	18.35 ± 0.27	13.94 ± 0.22	21.23 ± 0.74
	MIC/4	4.55 ± 0.04	—	6.1 ± 0.2
	MIC/8	—	—	—
*PA01*	MIC	23.4 ± 0.14	31.4 ± 0.47	—
	MIC/2	9.5 ± 0.03	15.3 ± 0.25	—
	MIC/4	—	4.1 ± 0.02	—
	MIC/8	—	—	—

#### 3.1.3 QSI Zones in *C. violaceum* CV026

**FIGURE 4:**
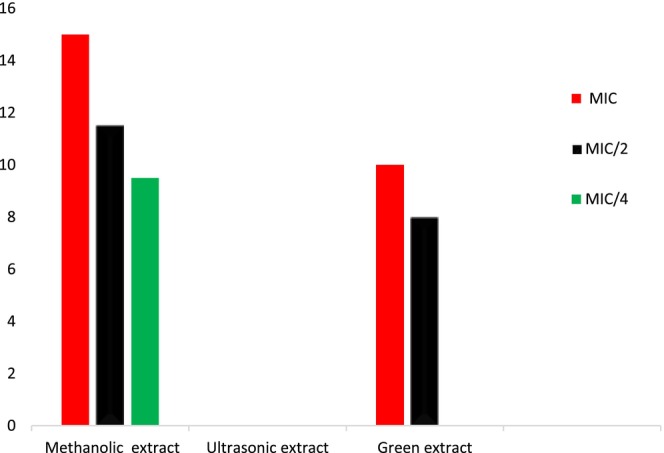
Quorum sensing inhibition zones in *Chromobacterium violaceum* CV026 of methanol, ultrasonic and green extracts of *Salvia triloba*.

Evaluation of QSI by green, ultrasonic‐assisted, and methanolic extracts of *S. triloba* was carried out using the *C. violaceum* CV026 strain, with results presented in Figure 4. Before evaluating QSI, the MICs for all extracts against the selected strain were assessed. The MIC values recorded for the methanolic, ultrasonic‐assisted, and green extracts of *S. triloba* against *C. violaceum* CV026 were 0.5, 0.5, and 0.25 mg/mL, respectively. Among the examined extracts, the methanolic extract exhibited the most significant anti‐QS activity, as evidenced by inhibition zones of 15.0 ± 0.9, 11.5 ± 0.1, and 9.0 ± 0.3 mm at concentrations corresponding to MIC, MIC/2, and MIC/4, respectively. In contrast, the green extract showed anti‐QS activity with inhibition zones of 10.5 ± 0.5 and 8.0 ± 0.2 mm at MIC and MIC/2 concentrations, respectively. No QSI activity was observed for the ultrasonic‐assisted extract. Consequently, the potential for QSI was ranked as follows: methanolic extract > green extract > ultrasonic‐assisted extract.

#### 3.1.4 Inhibition of Violacein Production

**FIGURE 5:**
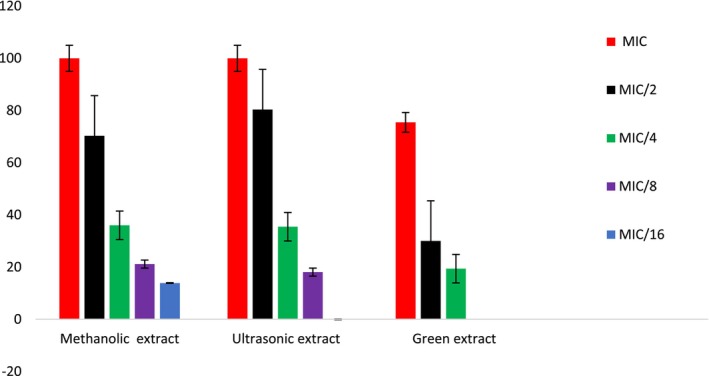
Violacein production inhibition in *Chromobacterium violaceum* CV12472 of methanol, ultrasonic and green extracts of *Salvia triloba*.

The methanolic extract, ultrasonic‐assisted extract, and green extract of *S. triloba* were subjected to AM activity utilizing *C. violaceum* CV12472 to evaluate inhibition of violacein production. Prior, the MIC values are measured, and they are implemented at concentrations of MIC and sub‐MIC. The results are described in Figure 5. The methanolic and ultrasonic‐assisted extracts showed considerable activity against *C. violaceum* CV12472 with 100% violacein inhibition at MIC values as 0.625 mg/mL. while green extract showed 75.5 ± 1.1% violacein inhibition activity against *C. violaceum* CV12472 at concentration of MIC 1.25 mg/mL. The inhibition of violacein production by methanolic extract at sub‐MIC concentrations was recorded as 70.3 ± 1.5%, 36.0 ± 0.7%, 21.2 ± 0.1% and 13.9 ± 0.1% at concentration of MIC/2, MIC/4, MIC/8, and MIC/16 respectively; in contrast, the violacein production inhibition by ultrasonic‐assisted extract recorded percentage inhibition as 80.4 ± 1.9%, 35.5 ± 0.8%, and 18.1 ± 0.5%, respectively, at concentrations of MIC/2, MIC/4, and MIC/8 respectively, while green extract exhibited percentage inhibition as 30.0 ± 0.5% and 19.4 ± 1.0% at concentration of MIC/2 and MIC/4, respectively. Therefore, the ability to inhibit violacein production could be classified as methanolic extract > ultrasonic‐assisted extract > green extract.

#### 3.1.5 Swarming Motility Inhibition

**FIGURE 6:**
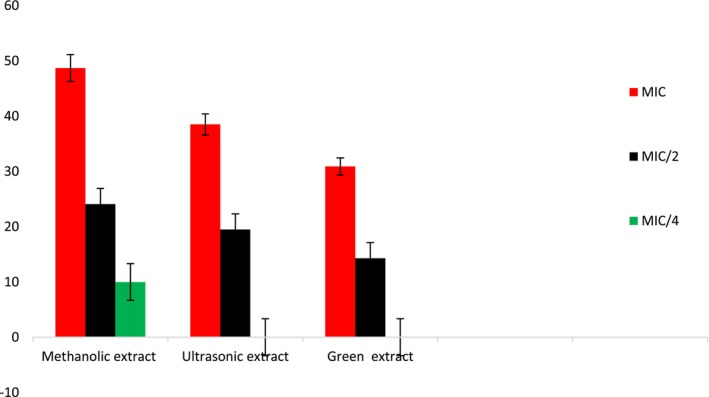
Swarming motility inhibition on *Pseudomonas aeruginosa* PA01 of methanol, ultrasonic and green extracts of *Salvia triloba*.

The green extract, methanolic extract, and ultrasonic‐assisted extract of *S. triloba* were assessed for their ability to inhibit swarming motility in *P. aeruginosa PA01* at both MIC and sub‐MIC concentrations, and the results are presented in Figure 6. The methanolic extract demonstrated the highest percentage of swarming motility inhibition, recording values of 48.7 ± 2.0% at MIC, 24.1 ± 0.3% at MIC/2, and 10.0 ± 0.5% at MIC/4. In comparison, the ultrasonic‐assisted extract showed inhibition rates of 38.5 ± 1.1% and 19.5 ± 0.7% at MIC and MIC/2 concentrations, respectively. Therefore, the effectiveness of inhibition can be classified as follows: methanolic extract > ultrasonic‐assisted extract > green extract.

### 3.2 Enzyme Inhibition Activities

#### 3.2.1 Anticholinesterase Activity

**FIGURE 7:**
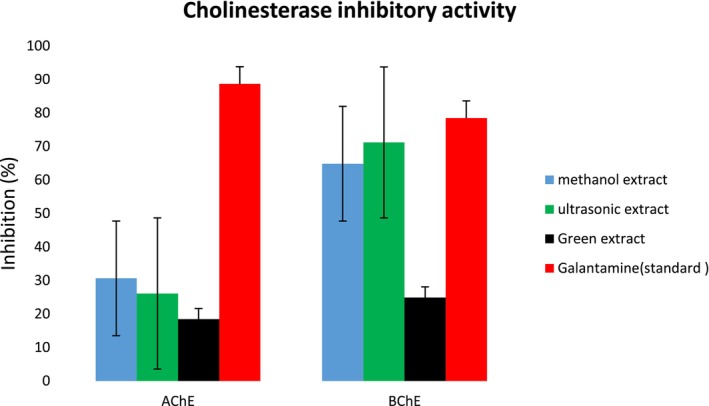
Butyrylcholinesterase (BChE) inhibition of methanolic extract, ultrasonic extracts, and green extract of *Salvia triloba*.

The capacity of the methanol extract, ultrasonic‐assisted extract, and green extract of *S. triloba* to inhibit specific enzymes, including AChE and BChE, was evaluated in vitro, as indicated by the reported results in Figure 7. Methanol extract and ultrasonic‐assisted extract exhibited very moderate percentage inhibition (64.85 ± 1.14% and 71.19 ± 0.89%) against BChE with IC_50_ = 102.3 ± 1.20 and 74.80 ± 0.98 μg/mL, respectively, while recorded low activity against AChE with percentage inhibition 30.63 ± 0.54% and 26.11 ± 0.71%, respectively. In contrast, the green extract showed weak inhibition (24.89 ± 0.19% and 18.44 ± 0.73%) against BChE and AChE, respectively. Therefore, the order of potential cholinesterase enzyme inhibition could be in the order of ultrasonic extract > methanol extract > green extract.

#### 3.2.2 Anti‐Urease Activity

**FIGURE 8:**
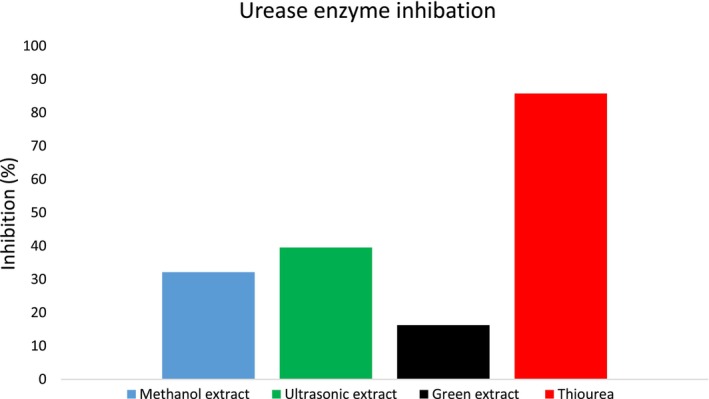
Urease enzyme inhibition of methanolic extract, ultrasonic extracts, and green extract of *Salvia triloba*.

The current study investigated the urease inhibition abilities of methanolic, ultrasonic, and green extracts from *S. triloba*. The results are presented in Figure 8. The findings revealed that the ultrasonic‐assisted, methanolic, and green extracts showed limited activity, with percentage inhibitions of 39.51 ± 0.58%, 32.12 ± 0.76%, and 16.24 ± 0.28%, respectively.

### 3.3 Antioxidant Activities

#### 3.3.1 β‐Carotene‐Linoleic Acid Assay

**FIGURE 9:**
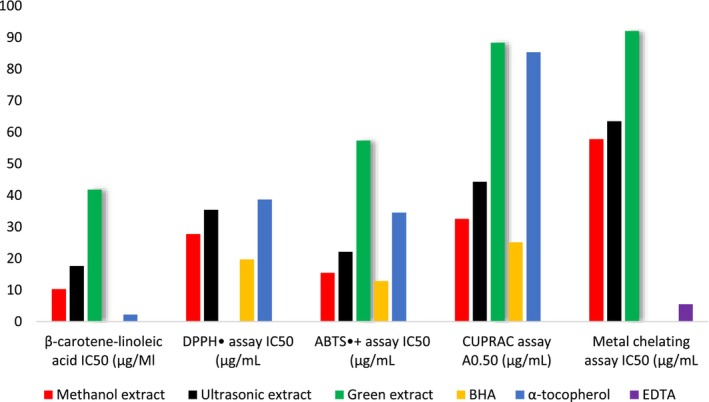
Antioxidant activity (IC_50_ μg/mL) of methanolic extract, ultrasonic extracts, and green extract of *Salvia triloba* evaluated by different methods.

The ability to inhibit lipide peroxidation by methanol extract, ultrasonic‐assisted extract, and green extract of *S. triloba* was estimated. The obtained results are shown in Table 4 and Figure 9. The methanolic extract and ultrasonic‐assisted extract showed significant inhibition with (IC_50_ = 10.29 ± 0.36 and 17.66 ± 0.74 μg/mL), respectively, while green extract exhibited moderate lipide peroxidation inhibition with (IC_50_ value 41.80 ± 0.92 μg/mL). Hence, the lipid peroxidation inhibition activity could be ranked as methanolic extract > ultrasonic‐assisted extract > green extract.

**TABLE 4:** Antioxidant activity of the methanol, ultrasonic‐assisted and green extracts of *S. triloba* by the β‐carotene‐linoleic acid, DPPH^•^, ABTS^•+^, CUPRAC, and metal‐chelating assays^a^.

Antioxidant activity					
Extracts	β‐Carotene‐linoleic acid assay IC_50_ (μg/mL)^a^	DPPH^•^ assay IC_50_ (μg/mL)	ABTS^•+^ assay IC_50_ (μg/mL)	CUPRAC assay *A*_0.5_ (μg/mL)^b^	Metal chelating assay IC_50_ (μg/mL)
Methanol extract	10.29 ± 0.36	27.77 ± 0.55	15.49 ± 0.95	32.54 ± 0.84	57.80 ± 0.95
Ultrasonic extract	17.66 ± 0.74	35.43 ± 0.93	22.14 ± 0.17	44.30 ± 0.77	63.51 ± 0.71
Green extract	41.80 ± 0.92	> 100	57.38 ± 0.88	88.34 ± 0.72	92.08 ± 0.65
BHA^c^	1.46 ± 0.03	19.70 ± 0.25	12.85 ± 0.52	25.12 ± 0.01	NT
α‐Tocopherol^c^	2.25 ± 0.04	38.70 ± 0.32	34.50 ± 0.48	85.36 ± 0.02	NT
EDTA^c^	NT	NT	NT	NT	5.51 ± 0.53

Abbreviation: NT, not tested.

^a^ IC_50_ values represent the means ± SEM of three parallel measurements (*p* < 0.05).

^b^ *A*_0.50_ values represent the means ± SEM of three parallel measurements (*p* < 0.05).

^c^ Reference compounds.

#### 3.3.2 DPPH ^•^ Assay

The free radical scavenging activity of each green extract, ultrasonic‐assisted extract, and methanol extract of *S. triloba* was evaluated as the results labeled in Table 4 and Figure 9. The methanolic extract and ultrasonic‐assisted extract exhibited significant free radical scavenging activity (IC_50_ = 27.77 ± 0.55 and 35.43 ± 0.93 μg/mL). Both these extracts exhibited better activity than the standard α‐tocopherol (IC_50_ = 38.70 ± 0.32 μg/mL); in contrast, no activity was absorbed by green extract. Therefore, the DPPH radical scavenging capacity of the extract tested could classified as methanolic extract > ultrasonic‐assisted extract > green extract.

#### 3.3.3 ABTS ^•+^ Assay

Table 4 describes the results of the assessment of antioxidant activity by utilizing ABTS^•+^ assay of methanol extract, ultrasonic‐assisted extract, and green extract from *S. triloba*. The considerable ABTS^·+^ antioxidant activity was shown via the methanolic extract with IC_50_ = 15.49 ± 0.95 μg/mL followed by ultrasonic extract with IC_50_ = 22.14 ± 0.17 μg/mL, while green extract showed moderate activity with IC_50_ = 57.38 ± 0.88 μg/mL. The considerable ABTS^·+^ antioxidant activity showed by methanol and ultrasonic‐assisted extracts were better than standard α‐tocopherol (IC_50_ = 34.50 ± 0.48 μg/mL) and close to the result showed by standard BHA (IC_50_ = 12.85 ± 0.52 μg/mL). Consequently, the order is ABTS^·+^ antioxidant activity as methanolic extract > ultrasonic‐assisted extract > green extract (Figure 9).

#### 3.3.4 CUPRAC Assay

The estimation results of CUPAC assay of methanol, ultrasonic‐assisted, and green extract of *S. triloba* are described in Table 4 and Figure 9. The results revealed that the methanolic extract and ultrasonic‐assisted extract had considerable activity (*A*_0.50_ values =32.54 ± 0.84 μg/mL) and (*A*_0.50_ values = 44.30 ± 0.77 μg/mL), respectively, while green extract exhibited moderate activity with *A*_0.50_ values = 88.34 ± 0.72 μg/mL. However, the results exhibited by methanol and ultrasonic‐assisted extract were found to be better than standard α‐tocopherol (*A*_0.50_ = 85.36 ± 0.02 μg/mL) and close to standard BHA (*A*_0.50_ = 25.12 ± 0.01 μg/mL), hence, the order CUPRAC antioxidant activity could be as methanolic extract > ultrasonic‐assisted extract > green extract.

#### 3.3.5 Metal Chelating Assay

Methanolic, ultrasonic‐assisted extracts as well as green extract of *S. triloba* were examined for their antioxidant activity using the metal chelating assay, and the results are reported in Table 4 and Figure 9. The results revealed the highest activity exhibited by methanol extract with IC_50_ = 57.80 ± 0.95 μg/mL followed by ultrasonic‐assisted extract with IC_50_ = 63.51 ± 0.71 μg/mL, while green extract exhibited activity with IC_50_ = 92.08 ± 0.65 μg/mL.

## 4 Discussion

The aim of the current study was to estimate the presence of various phenolic compounds of *S. triloba* via two types of extraction, green extract and conventional extract representative of methanol and ultrasonic‐assisted extracts. The amounts of phenolic compounds were found abundantly in each of the extract, although their amount varied. Sixteen compounds were present in the ultrasonic‐assisted extract: protocatechuic acid, catechin, chlorogenic acid, *p*‐hydroxy benzoic acid, caffeic acid, vanillin, rutin, myricetin, ellagic acid, rosmarinic acid, quercetin, luteolin, trans‐cinnamic acid, kaempferol, and apigenin were present in this extract. Among them, four compounds including trans‐cinnamic acid, *p*‐hydroxy benzoic acid, kaempferol, and vanillin were not found in methanol extract but were found in ultrasonic‐assisted extract; on the other hand, thirteen phenolic compounds were detected in green extract including protocatechuic acid, gallic acid, caffeic acid, chlorogenic acid, *p*‐hydroxy benzoic acid, vanillin, rutin, ellagic acid, myricetin, rosmarinic acid, luteolin, kaempferol, and apigenin. Among them, *p*‐hydroxy benzoic acid, vanillin, and kaempferol were not available in methanol extract. Importance of phenolic compounds as antioxidant agents is documented previously (Moo‐Huchin et al. 2015) as well as their possess the worth of diverse therapeutic properties such as antidiabetic, antiinflammatory, antiproliferative, antiaging agents (Shukitt‐Hale, Lau, and Joseph 2008).

As observed, the green extraction utilized demonstrated extraction efficiency of phenolic compounds almost similar to conventional extraction, and this is consistent with recent research findings that emphasize the green solvents potential as promising alternative to conventional solvents (Dai, Witkamp, et al. 2013; Paiva et al. 2014; Wei et al. 2015).

The results of antibacterial activity against the selected bacterial strains, which include *S. aureus*, *E. coli*, *C. albicans*, *E. faecalis*, and *P. aeruginosa* PA01, showed significant effectiveness especially by methanolic, extract of *S. triloba*. This compelled us for further investigation the potential inhibitory effects of certain extracts on biofilm formation, quorum‐sensing inhibition, violacein inhibition, and swarming motility inhibition.

Biofilms are complex communities of microorganisms that adhere to surfaces and are surrounded by a protective matrix. They are responsible for various infections and diseases, including dental plaque, urinary tract infections, and chronic wounds (Sharma et al. 2023). Several plants have been found to inhibit biofilm formation and disrupt existing biofilm such as cranberry, garlic, onion, and green tea (Hengge 2019; Laplante et al. 2012; Somrani, Debbabi, and Abidi 2020). Therefore, The use of natural sources as alternative medicine to inhibit the growth and formation of biofilms caused by pathogenic microorganisms is great significant. In current study, each extracts showed significant inhibition of biofilm formation. The results of current investigation are consistent with previous studies that discussed the ability of *S. triloba* crude and essential oil to inhibit biofilm formation (Mag et al. 2010). However, in this study, we used green solvent during extraction for the first time, and the results demonstrated the effectiveness of green extract as the biofilm formation inhibitor. The results presented in this investigation suggest that the disruption of the cell membrane, possibly induced by phenolic compounds at concentration below the MIC, may be responsible for the observed results (Burt et al. 2014). In addition, previous research has indicated that biofilm formation is influenced by several factors, such as, QS signaling (dos Reis Ponce‐Rossi et al. 2016). Nevertheless, the inhibition of QS likely plays a vital role in preventing biofilm formation. Future studies should focus on elucidating the mechanisms underlying the observed inhibition of biofilm formation in this study and other related studies.

Inhibiting QS holds significant importance as it has the potential to disrupt the communication and coordination between bacterial populations. QS serves as a mechanism by which bacteria can interact and synchronize their actions in response to changes in population density (Castillo‐juárez et al. 2015; Moreno‐Gámez 2023). This communication relies on the production and detection of signaling molecules known as autoinducers (Federle 2011). By hindering QS, it becomes possible to disrupt bacterial processes including biofilm formation, the production of virulence factors, and development of antibiotic resistance (Sionov 2022; Subhadra, Oh, and Choi 2016). Our investigation revealed that conventional extract (represent by methanolic extract) and the green extract of *S. triloba* showed significant QSI. In a recent study by Santos et al. (2021), the phenolic compounds were identified as potent anti‐QS agents (Santos et al. 2021). Furthermore, extensive research has consistently shown the remarkable inhibitory potential of these compounds on violacein production, particularly, Caffeic acid, which has been found to reduce violazine production via an impressive 75%, while Gallic acid exhibited a reduction of up to 59%. Oleuropine glycoside and Epicatechin also demonstrated inhibitory effects, reducing violazine production by 51%. Furthermore, Ferric acid exhibited a considerable inhibition up to 72%, while Floridine showed a reduction up to 48% (Borges, Saavedra, and Simões 2012; Chenia 2013); these findings highlight the significant anti‐QS properties of phenolic compounds, making them excellent candidates for further exploration in related fields. In the present study, all extracts demonstrated pronounced inhibition of violacein production, with the methanolic and ultrasonic‐assisted extracts exhibiting the most appreciable inhibition. Moreover, phenolic compounds such as caffeic acid and gallic acid were detected in each examined extracts. Therefore, we propose that the detected violacein production inhibition in current study may be attributed to the existence of these phenolic compounds acting either separately or in a synergistic manner. It should be noted that *Chromobacterium violaceum* CV12472 produces purple pigment violacein when growing normally while the mutant strain *C. violaceum* CV026 requires an externally supplied AHL (acylhomoserine lactone) to produce violacein. Violecein serves as an antioxidant protection and also as a signal molecule in pathogenic bacteria and helps to coordinate colony behavior as the virulence factor. Violecein is an easily measurable indication of QS activity, and both bacteria are used complementarily in which *C. violaceum* CV12472 indicates signal emission, while *C. violaceum* CV026 indicates signal reception (Popova et al. 2021; Talla et al. 2023). The results therefore reflect the potential of *S. triloba* extracts in inhibiting both signal emission and reception. As shown in Figure 5, there is an observable reduction in violacein production when MIC and sub‐MIC concentrations of *S. triloba* extracts are used. The QS inhibition zones correspond to the cream halos around the wells against the purple colored lawn of growing chromobacteria (Popova et al. 2021; Talla et al. 2023). It is observed that the percentage reduction in the violacein pigment production follows a concentration‐dependent trend.

Swarming motility is often associated with the formation of biofilm. Bacteria that exhibit swarming behavior are more likely to create robust biofilms, which offer protection against AM agents and the host's immune response (Chelvam, Chai, and Thong 2014). By inhibiting swarming motility, the initial stages of biofilm formation can be disrupted, making bacteria more susceptible to elimination by the immune system or AM treatments (Srinivasan and Santhakumari 2021). Consequently, inhibition of swarming motility may be a potential target for the development of AM strategies, as it can impede the spread and virulence of pathogenic bacteria. The methanolic extract, ultrasonic‐assisted extract, and green extract of *S. triloba* showed swarming motility inhibition and significant activity was detected by methanolic extract. Previous investigations have shown that phenolic compounds can inhibit swarming motility; for instance, Borges, Saavedra, and Simões (2012) reported that the phenolic compounds such as ferulic acid, gallic acid, and caffeine could inhibit the swarming motility of several bacteria comprising *S. aureus*, *P. aeroginosas*, *L. monocytogenes*, and *E. coli* (Borges, Saavedra, and Simões 2012). These results highlight the considerable potential of phenolic compounds as inhibitors of swarming motility. It was noted that all the extracts analyzed in the current study contained ferulic acid, gallic acid, and caffeine, suggesting that the occurrence of these compounds may contribute to the extracts' ability to inhibit swarming motility. As far as we know, the current study is the first investigation to demonstrate the efficacy of *S. triloba* in suppressing swarming motility. Figure 6 indicates the decrease in the *P. aeruginosa* PA01 swarm fronts when MIC and sub‐MIC amounts of the *S. triloba* (extracts) are used. It is observed that the percentage reduction in the swarm areas covered by the bacterial motility reduces in a concentration‐dependent manner.

The ability of *S. triloba* extracts including methanolic, ultrasonic‐assisted extracts, and green extract were examined for the first time to inhibit AChE and BChE as well as urease enzymes. Methanolic extract and ultrasonic‐assisted extract showed moderate activity, while green extract revealed weak activity against both (AChE and BChE) enzymes. Numerous studies have confirmed ability of natural products and their derivatives to cure Alzheimer's disease via inhibiting the enzymes (AChE and BChE); for instance, the essential oil obtained from *C. officinalis*, *Origanum syriacum*, and *Salvia lavandulaefolia* have been reported to exhibit considerable anticholinesterase activity in previous investigation (Ak et al. 2021; Loizzo et al. 2009; Perry et al. 2000); furthermore, in previous investigation, the phenolic compounds have been documented as effective inhibitors of both AChE and BChE enzymes (Roseiro, Rauter, and Serralheiro 2012). Moreover, the phenolic compounds found in certain Salvia species have shown significant inhibition of both AChE and BChE enzymes (Kocakaya et al. 2020). Consequently, the (AChE and BChE) inhibition detected in current study may be attributed to present phenolic compound identified already in extracts. Meanwhile, the results from the urease inhibition assay indicated that the methanolic and ultrasonic‐assisted extracts exhibited moderate urease inhibition, while the green extract showed only slight inhibition against the urease enzyme. Previous studies have reported that phenolic compounds such as gallic acid and quercetin have a significant capacity to inhibit the urease enzyme (Svane et al. 2020), Although these compounds were present in all tested extracts, the green extract's minimal inhibition could be due to the state of the extract. In an extract, compounds can interfere with each other, potentially masking the activity of one compound by another in the mixture.

Antioxidant molecules play a vital role in reducing the risk of chronic diseases such as cancer and heart disease, which are linked to oxidative stress caused by free radicals in our bodies. These free radicals can damage cells and, in some cases, contribute to cell death (Kurutas 2016; Willcox et al. 2010). The body's systems are protected from the harmful effects of oxidative stress by antioxidant substances that prevent and scavenge free radicals (Hajam et al. 2022; Sharifi‐Rad et al. 2020). In the current study, antioxidant activity was evaluated using several assays, including the DPPH^•^ assay, β‐carotene‐linoleic acid assay, ABTS^•+^ assay, metal chelating assay, and CUPRAC assay. The free radical quenching capacity of *S. triloba* extracts, including methanolic extract, ultrasonic‐assisted extract, and green extract, was assessed using DPPH and ABTS assays. The DPPH is a stable‐free radical that can be easily quenched by an antioxidant agent, losing its absorption by accepting an electron or a radical species. (Wang, Wang, and Li 2013). Our investigation revealed bot methanolic extract and ultrasonic‐assisted extract exhibited considerable in scavenging‐free radical. In DPPH assay; even highest than the standard used (α‐tocopherol). In ABTS assay, we found that the all extracts tested showed the ABTS radical scavenging capacity; furthermore, the methanol and ultrasonic‐assisted extracts exhibited capacity better than standard used (α‐tocopherol). In addition, the antioxidant ability also evaluated by using the carotene/linoleic acid bleach test, the activity test for neutralizing free linoleate radicals and free radicals that are formed in the system and attack highly unsaturated β‐carotene patterns (Barros et al. 2009). The results obtained from current study showed that every extracts tested possessed the activity. The activity could be attributed to presence phenolic compounds in extracts which described previously as antioxidant agents whereat could inhibit the spread of β‐carotene degradation by linoleate or other radicals that might be formed in the system. The capacity of *S. triloba*, extracts including methanolic, ultrasonic‐assisted, and green extracts to reduce Cu^2+^ to Cu^1+^ were evaluated. All extracts tested revealed ability to reduce Cu^+2^; however, the methanolic and ultrasonic‐assisted extracts exhibited the greater capacity even than reference used (α‐tocopherol). The ability of antioxidants to chelate ferrous ions was assessed to determine the metal chelation capacity. In this study, the ferrous ion chelating capacity of methanol, ultrasonic‐assisted, and green extracts of *S. triloba* were determined utilized the Fe(II) ferrozine test system, with results reported as EDTA equivalents. The results showed that methanolic extract had the highest chelating activity among the studied extracts, followed by the ultrasonic and green extracts. The results of current study on antioxidant activity are consistent with previous studies on *S. triloba* (Özüpek, Pekacar, and Orhan 2023) and *Salvia* species, including *Salvia fruticose* (Boukhary et al. 2016), *Salvia virgata* Jacq (Karatoprak, Ilgün, and Koşar 2016), *Salvia verticillata* (Tosun et al. 2009), and *Salvia officinalis* L (Hamrouni‐Sellami et al. 2013), indicating significant antioxidant properties. Phenolic compounds were found abundantly in all the extracts; therefore, the antioxidant capacity found in the current study may be related to the presence of phenolic compounds confirmed as antioxidant agents such as: gallic acid (Fernandes and Salgado 2016), quercetin (Xu et al. 2019), and rosmarinic acid (Adomako‐Bonsu et al. 2017).

We reported the ability green solvents to extract bioactive compounds from *S. sclarea* in previous studies and demonstrated significant biological activities (Quradha et al. 2024), Herein, we also confirm the effectiveness of green solvents in extracting potent compounds from natural sources.

## 5 Conclusions

Based on the results mentioned earlier, both conventional and green solvents showed almost similar yields in identifying phenolic compounds. Rosmarinic acid was found in high concentrations in both type of extracts, while trans‐cinnamic acid was only existing in ultrasonic extract. The green extraction demonstrated superior biofilm inhibition compared to the conventional solvent for *E. faecalis*. The methanolic extract exhibited superior biofilm inhibition than ultrasonic and green extracts against *E. coli* and *S. aureus* at MIC concentration. Furthermore, both methanolic extract and green extract showed significant inhibition of QS against *C. violaceum* CV026. Both conventional and green extracts exhibited notable antioxidant activity, with the highest activity detected by the methanolic extract based on various assays: β‐carotene‐linoleic acid with IC_50_ = 10.29 ± 0.36 μg/mL, DPPH^•^ with IC_50_ = 27.77 ± 0.55 μg/mL, ABTS^•+^ with IC_50_ = 15.49 ± 0.95 μg/mL, metal chelating with IC_50_ = 57.80 ± 0.95 μg/mL, and CUPRAC with *A*_0.50_ = 32.54 ± 0.84 μg/mL. This study demonstrated the ability of green solvents to extract bioactive compounds from plants and exhibited similar or close biological activities potential exhibited by conventional solvents. Consequently, the study recommended more investigations on green solvents and explore its effectiveness in replacing conventional solvents and their potential use in extracting bioactive compounds from natural sources.

## Author Contributions

**Alfred Ngenge Tamfu:** methodology (equal), writing – original draft (equal). **Selcuk Kucukaydin:** methodology (equal), validation (equal). **Mudassar Iqbal:** data curation (equal), methodology (equal), writing – original draft (equal). **Rasool Khan:** conceptualization (equal), writing – review and editing (equal). **Ozgur Ceylan:** methodology (equal), supervision (equal). The main experiments, including sampling in landfall sites, synthesis of green solvents, and optimization of organic and green solvents, write the draft article was performed by (Mohammed Mansour Quradha). while (Mehmet Emin Duru) provided supervision and project administration. (S.K) performed enzyme inhibition and antioxidant investigation, Abdulkader Moqbel Farhan Qahtan is contributed to writing ‐ review and editing of manuscript.

## Conflicts of Interest

The authors declare no conflicts of interest.

## Data Availability

All data generated or analyzed in current study are included in the published article.
